# Low-Tech, Pilot Scale Purification of a Recombinant Spider Silk Protein Analog from Tobacco Leaves

**DOI:** 10.3390/ijms17101687

**Published:** 2016-10-09

**Authors:** René Heppner, Nicola Weichert, Angelika Schierhorn, Udo Conrad, Markus Pietzsch

**Affiliations:** 1 Department of Downstream Processing, Institute of Pharmacy, Faculty of Sciences I—Biosciences, Martin Luther University Halle-Wittenberg, Weinbergweg 22, Halle 06120, Germany; rene.heppner@pharmazie.uni-halle.de; 2 Institute of Plant Genetics and Crop Plant Research—IPK, Corrensstrasse 3, Seeland OT Gatersleben 06466, Germany; weichert@ipk-gatersleben.de (N.W.); conradu@ipk-gatersleben.de (U.C.); 3 Institute of Biochemistry and Biotechnology, Faculty of Sciences I—Biosciences, Martin Luther University Halle-Wittenberg, Kurt-Mothes-Str. 3, Halle 06120, Germany; angelika.schierhorn@biochemtech.uni-halle.de

**Keywords:** spider silk protein variants, purification, pilot scale, downstream processing, scale up

## Abstract

Spider dragline is used by many members of the Araneae family not only as a proteinogenic safety thread but also for web construction. Spider dragline has been shown to possess high tensile strength in combination with elastic behavior. This high tensile strength can be attributed to the presence of antiparallel β-sheets within the thread; these antiparallel β-sheets are why the protein is classified as a silk. Due to the properties of spider silk and its technical and medical uses, including its use as a suture material and as a scaffold for tissue regeneration, spider dragline is a focus of the biotechnology industry. The production of sufficient amounts of spider silk is challenging, as it is difficult to produce large quantities of fibers because of the cannibalistic behavior of spiders and their large spatial requirements. In recent years, the heterologous expression of genes coding for spider silk analogs in various hosts, including plants such as *Nicotiana tabacum*, has been established. We developed a simple and scalable method for the purification of a recombinant spider silk protein elastin-like peptide fusion protein (Q-/K-MaSp1-100× ELP) after heterologous production in tobacco leaves involving heat and acetone precipitation. Further purification was performed using centrifugal Inverse Transition Cycling (cITC). Up to 400 mg of highly pure spider silk protein derivatives can be isolated from six kilograms of tobacco leaves, which is the highest amount of silk protein derivatives purified from plants thus far.

## 1. Introduction

Orb weaving spiders, such as *Nephila clavipes*, produce up to seven different types of silk [1,2]. One of these spider silk proteins, the Major Ampullate Spidroin 1 (MaSp1), is a 275 kDa protein of known sequence that provides strength to parts of the web [3]. Spiders use this type of silk, which is also known as dragline silk, as a frame and safety thread. The structural motifs in dragline silk responsible for its strength are blocks of antiparallel-oriented beta-sheets consisting of poly-alanine and/or poly-glycine blocks. These blocks are embedded in an amorphous matrix comprised of 3_10_ helices, which consist of repeating GlyGlyX (X = Tyr, Leu, Gln) motifs [4,5]. As much as 90% of a silk thread can consist of these two motifs [6]. The reasons for researchers′ desire to uncover the secrets of dragline silk are obvious: dragline silk is: (1) a natural product; (2) produced under ambient conditions; and (3) possesses a strength on the same order of magnitude as the strongest man-made fiber (Kevlar^®^; DuPont de Nemours, Neu-Isenburg, Germany) [7].

The recombinant production of spider silks has gained increasing interest. Possible areas of application for spider silk include lightweight construction, bulletproof vests or clinical uses [8]. Today, many interesting proteins are produced using fast-growing genetically engineered microorganisms such as bacteria and yeast. However, the production of spider silk analogs in bacteria and yeast is challenging: these organisms are unable to generate a sufficient amount of the amino acids glycine and alanine, the main constituents of the capture spiral core protein Flag (ca. 54% Gly, and 8% Ala) and MaSp1 (ca. 42% Gly, and 26% Ala) [4,9]. Moreover, the high number of repetitive elements in spider silk proteins results in translational errors. As a result, recombinant proteins produced in these organisms are often truncated [10,11,12]. In a recent work, the aforementioned problems were overcome in a process that yielded 1 g/L of a high molecular weight (285 kDa) spider silk analog [12]. Other possible hosts for the production of recombinant spider silk proteins are plants such as *Nicotiana tabacum*. *Nicotiana tabacum* is generally recognized as safe (GRAS) with no risk of cross-contaminating the food chain (Available online: http://www.fda.gov/Food/IngredientsPackagingLabeling/GRAS/; accessed on 18 August 2016). *Nicotiana tabacum* has already been used for the production of antibodies, vaccines and spider silk analogs [13,14,15,16]. Plant-based expression systems are able to provide complex, correctly folded and post-translationally modified proteins [17]. Relatively low cost, safety and scalability are specific advantages of plant expression systems [18].

**Figure 1:**
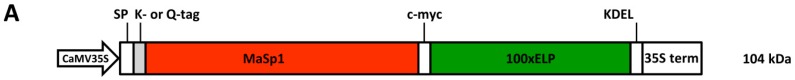

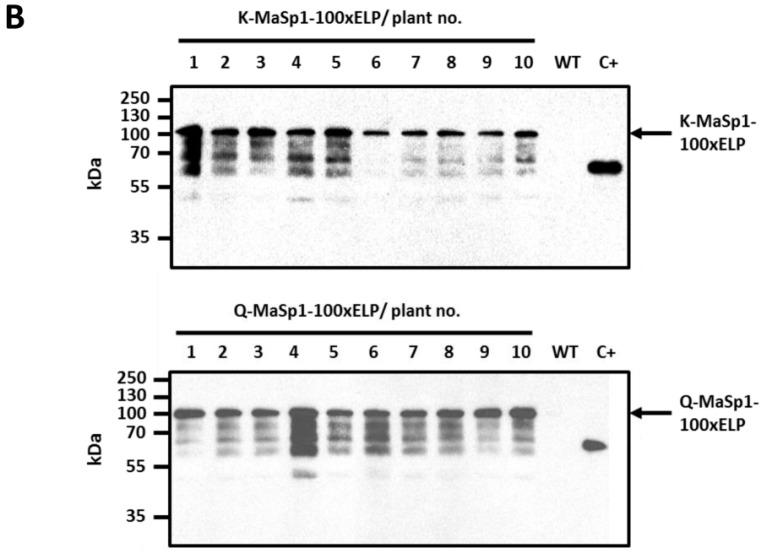
Expression of ELPylated Major Ampullate Spidroin 1 (MaSp1) fusion proteins in tobacco leaves. (**A**) Schematic representation of the plant expression cassette for the expression of MaSp1-100× ELP monomers. CaMV35S: *Cauliflower Mosaic Virus* 35S promoter; SP: legumin B4 signal peptide; K- or Q-tag: lysine or glutamine tag; 100× ELP: 100 repeats of elastin-like pentapeptide VPGXG (V:A:G = 5:2:3); KDEL: ER retention sequence; c-myc: immunodetection tag; 35S term: *Cauliflower Mosaic Virus* 35S terminator; (**B**) Leaf extracts of transgenic tobacco plants accumulating either K-MaSp1-100× ELP or Q-MaSp1-100× ELP protein and the corresponding wild type cultivar *Nicotiana tabacum* cv. Samsun NN separated by SDS-PAGE (12% PAA). MaSp1 fusion protein monomers were immunodetected by Western blotting based on the c-myc tag. c-myc standard c+: 2 ng of anti-TNF-VHH-100× ELP [25].

The main cost of recombinant protein production is incurred in the purification steps; these steps can account for up to 80% of the total production costs [19]. Such high purification costs typically result from the use of costly chromatographic materials and time-consuming purification steps. Separating a target protein from contaminants can be promoted by covalent attachment of an additional purification tag [20]. Such tags usually have a high affinity to specific ligands, a property that is then exploited during purification. In 1999, an alternative purification method was developed that allows expensive chromatographic purification steps to be replaced with easy and cheap precipitation steps. It was found that a tandem repeat of ValProGlyXGly (X = Val:Ala:Gly = 5:2:3) called an elastin-like polypeptide (ELP) exhibited an agglomeration/resolubilization behavior that could be triggered during downstream processing via shifts in ionic strength and/or temperature. The purification method based on this behavior is called Inverse Transition Cycling (ITC) [21]. After induced agglomeration, target proteins can be separated either via centrifugation (cITC) or a membrane-based (mITC) procedure [2,3,4,5,6,7,8,9,10,11,12,13,14,15,16,17,18,19,20,21,22,23,24]. The spider silk analogs that are the focus of the present investigation contain such an ELP tag (see Figure 1A) and were previously purified via mITC [16].

Scale-up of the mITC procedure turned out to be extremely challenging. First, during the initial incubation step, a large volume has to be tempered and processed; Second, micron-scale particles need to be separated from soluble contaminants at elevated temperatures (usually 40 °C), followed by resolubilization at low temperatures; Third, membrane filters are easily fouled and cannot be reused; Finally, there is considerable adsorption of the target protein on membranes, which leads to a reduced yield. To overcome these difficulties, we developed a cheap, robust and scalable low-tech purification procedure based on solvent precipitation and centrifugation.

## 2. Results

Tobacco leaves producing either Q-MaSp1-100× ELP or K-MaSp1-100× ELP (target proteins, TPs) in the Endoplasmic Reticulum were used to develop a pilot scale strategy for the purification of MaSp1 fusion proteins (see Figure 1A for the design of the protein and Ref. [16] for details). Briefly, a Major Ampullate Spidroin 1 derived gene (MaSp1), originated from the appropriate *Nephila clavipes* sequence, was designed to allow in vitro post-translational multimerization by a microbacterial transglutaminase. ELP was applied at C-terminal site of MaSp1 for both enhancing expression and purification. The resulting cassettes K-MaSp1-100× ELP (lysine-tagged) and Q-MaSp1-100× ELP (glutamine-tagged) encoded ELPylated spider silk fusion proteins and were used to stably transform tobacco plants. Two transgenic lines were selected out of 16 (Q-MaSp1100× ELP) and 24 (K-MaSp1-100× ELP) recombinant protein expressing T_0_ plants. Western blotting analysis of these plants showed the expected size of 104 kDa [16]. The offspring of these plants was used in the experiments described in the actual paper. Target protein expression profiles were tested in vitro and controlled in greenhouse plants after 13 weeks of growth by Western blot analysis (Figure 1B). Plant material was harvested after 15 weeks of growth in the greenhouse. Purification was carried out at small (50 g), medium (300 g), and pilot (5–6 kg) scales and included: (i) heat treatment; (ii) cell disruption; (iii) acetone precipitation and (iv) cITC.

### 2.1. Post-Harvest Treatments of Plant Biomass—Target Protein Degradation Could Be Avoided

**Figure 2:**
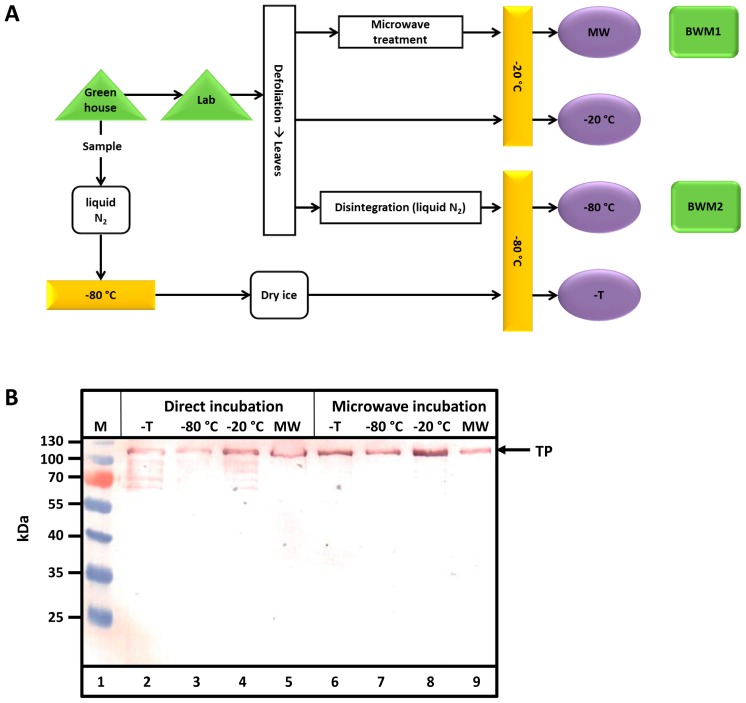
Prevention of post-harvest degradation of Q-MaSp1-100× ELP (TP) by microwave treatment. (**A**) Schematic of tobacco leaf processing after harvest. Bio wet mass 1 (BWM1) and bio wet mass 2 (BWM2) were used for the scale up experiments. Samples named MW, −20 °C, −80 °C and −T were either directly analyzed by SDS-PAGE and Western blotting (direct incubation) or after incubation in a microwave (microwave incubation); (**B**) Western blot of samples after different treatments and storage. After harvesting, a sample of tobacco leaves containing the TP was frozen using liquid nitrogen (−T; lanes 2 and 6). Residual plants were defoliated at 4 °C in the laboratory. One part was then frozen, mashed in liquid nitrogen, and stored at −80 °C (−80 °C; lanes 3 and 7), generating BWM1. Residual bio wet mass was frozen at −20 °C either with incubation in a microwave (MW; lanes 5 and 9; BWM2) or without (−20 °C; lanes 4 and 8). The arrow indicates the molecular size of the target protein (TP).

After harvest, defoliated tobacco leaves (bio wet mass, BWM) were handled in various ways (Figure 2A). Samples taken in the greenhouse were either transported to the lab or directly frozen in liquid nitrogen. Transport lasted 60 min at an ambient temperature (16 °C). Samples were either treated in a microwave or disintegrated by milling in liquid nitrogen before storage at −20 or −80 °C. Then, samples were either directly analyzed by sodium dodecyl sulfate-polyacrylamide gel electrophoresis (SDS-PAGE) and Western blotting (direct incubation) or after incubation in a microwave. Leaves at the small (50 g BWM1) and medium (300 g BWM2) scales were used in pilot scale (5–6 kg) experiments. As observed from the Western blots in Figure 2B, only samples heated in the microwave exhibited no TP degradation. Proteolytic degradation clearly occurs in all other cases.

### 2.2. Cell Disruption—Comparable Results in Quality and Amount After Upscaling

To determine the optimal cell disruption time, a suspension of microwave-treated biomass (see Materials and Methods) was prepared as described and homogenized either in a Waring blender (small and medium scale) or in a pilot-scale tabletop cutter. Samples were taken at various intervals and analyzed by SDS-PAGE. Silver staining was used to visualize spider silk bands. 

**Figure 3:**
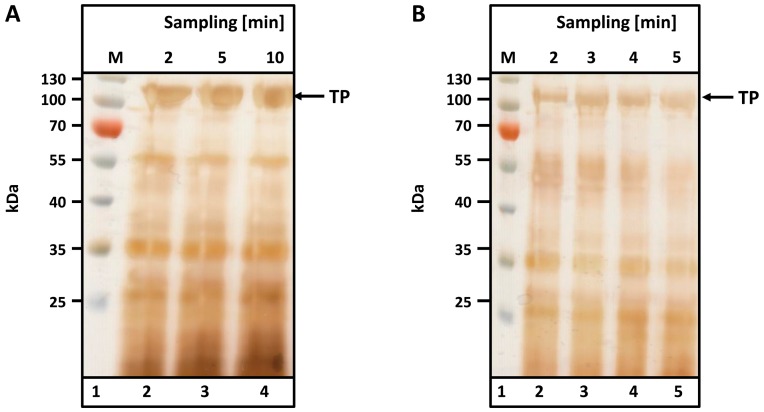
Release of K-MaSp1-100× ELP during cell disruption using: (**A**) a Waring blender; and (**B**) a table top cutter. *Nicotiana tabacum* leaves were incubated in a microwave and then stored at −20 °C. (**A**) Three hundred grams BWM2 were thawed (16 h, 4 °C), mixed with 450 mL tap water (4 °C) and sheared in a Waring blender for 10 min; (**B**) Five kilograms leaves in 6 L tap water (4 °C) were disintegrated using a table top cutter for 5 min. Samples were taken at the indicated time points. After centrifugation, 500 µL of the supernatant was dried and resolubilized in 200 µL SDS sample buffer. M-Prestained protein ladder. The arrow indicates the molecular size of the target protein (TP).

As observed from the SDS-PAGEs in Figure 3, no differences in protein content or band intensity were observed between the two different disruption machines used to disintegrate 300 g (Figure 3A, Waring blender) and 5 kg (B, table top cutter) of leaves. Complete disintegration is achieved after just 2 min in the Waring blender (Figure 3A, lane 2); no additional protein is liberated after 10 min (Figure 3A, lane 5). Similar results were observed with the table top cutter after 3 min of disruption. The TP is visible at the expected size (104 kDa), and major contaminants are visible below 60 kDa.

Mean particle sizes were analyzed using a laser diffraction particle size analyzer (LS13 320; Beckman Coulter GmbH, Krefeld, Germany) to compare the disruption methods. After 5 min, the Waring blender produced a mean particle size of 1.8 µm. A comparable particle size (1.5 µm) was generated by the tabletop cutter within 5 min.

### 2.3. Purification by Acetone Precipitation—Recovery of Target Proteins and Removal of Host Cell Proteins

**Figure 4:**
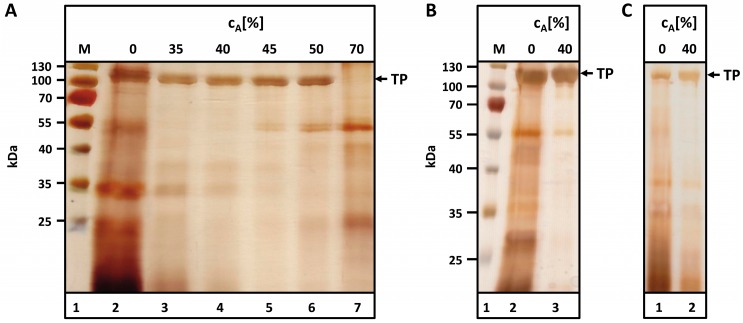
Purification of Q-MaSp1-100× ELP by fractionating acetone precipitation at: (**A**) small scale; (**B**) medium scale; and (**C**) pilot scale. (**A**) Fifty grams BWM1 was incubated in 150 mL disruption buffer at 90 °C for 1 h. After cooling to 4 °C and cell disruption using a Waring blender (10 × 30 s), the acetone concentration in the solution was increased stepwise; (**B**) Three hundred grams BWM2 were thawed for 16 h at 4 °C. Following addition of 450 mL tap water, cells were disintegrated in a Waring blender (5 min), and the suspension was mixed with acetone to a final concentration of 40% (*v*/*v*). After incubation (4 °C, 30 min) the soluble fraction (“40”) was separated from cell debris and precipitated; (**C**) Five thousand two hundred grams BWM2 were thawed for 16 h at 4 °C. After addition of 5 L tap water, cells were disintegrated in a table top cutter for 5 min. The suspension was clarified by filtration and centrifugation. The supernatant (“0”) was then incubated with acetone (*c*_A_ = 40%, 4 °C, 30 min), and the soluble fraction (“40”) was separated from cell debris and precipitated by centrifugation. Samples were dried and resolubilized in SDS buffer. M-Prestained protein ladder; *c*_A_: acetone concentration (*v*/*v*). The arrow indicates the molecular size of the target protein (TP).

A fractionating acetone precipitation was performed to separate the MaSp1-100× ELP fusion protein from host cell proteins (HCPs). Acetone was added to the centrifuged, particle-free protein solution after cell disruption. Precipitation behavior was first investigated at the small scale. As observed in Figure 4A, the TP remains soluble at acetone concentrations up to 50% (*v*/*v*).

Despite heat incubation, some HCPs remained soluble in the cell extract in addition to the TP (Figure 4A, lane 2). At an acetone concentration of 40% (*v*/*v*), most of those contaminants precipitated and could be separated via centrifugation. The TP was soluble up to 50% acetone (*v*/*v*) and precipitated at 70% acetone (*v*/*v*) (Figure 4A, lanes 5–7). Two major contaminants remained soluble at both 50% (*v*/*v*) and 70% (*v*/*v*) acetone (Figure 4A, lanes 5–7, bands at ~55 and 25 kDa).

To isolate larger amounts of the TP, the small scale experiment was scaled up to medium and pilot scales. For a simple and cost-effective process, the disruption buffer was replaced with tap water. To reduce the volumes that need to be handled, the solvent/biomass ratio was reduced from 1:3 (BWM:buffer, (*w*/*v*); 50 g BWM) to 1:1.5 (BWM:water, (*w*/*v*); 300 g BWM) and 1:1 (BWM:water, (*w*/*v*), 5–6 kg). Precipitation behavior was found to remain almost unchanged compared to pre-experiments (Figure S1).

The final fractionating precipitation process included the following steps: (i) addition of acetone to a final concentration of 40% (*v*/*v*) to the particle-free supernatant after cell disruption and centrifugation; (ii) removal of precipitated HCPs by centrifugation; (iii) addition of acetone to a final concentration of 70% (*v*/*v*) and (iv) centrifugation and recovery of the precipitated TP.

Precipitation behavior observed at the medium and pilot scales was essentially equivalent to the behavior observed at small scale (Figure 4B,C).

The most challenging aspect of the scale-up to pilot scale (10–12 L) was the solid-liquid separation after cell disruption. Centrifugation using a high-speed batch centrifuge was replaced by a sequence of: (i) filtration using a hydro press, which is usually used for juice clarification; and (ii) centrifugation using a semi-continuous tubular centrifuge. Solid-liquid separation during fractionating acetone precipitation was also performed using a tubular centrifuge.

The precipitation procedure developed for K-MaSp1-100× ELP was also used for the purification of Q-MaSp1-100× ELP. From 5.2 and 6.0 kg tobacco leaves, 37 g K-MaSp1-100× ELP and 67 g of Q-MaSp1-100× ELP were obtained, respectively.

### 2.4. Centrifugation-Based Inverse Transition Cycling—Successful Removal of Host Cell Proteins

**Figure 5:**
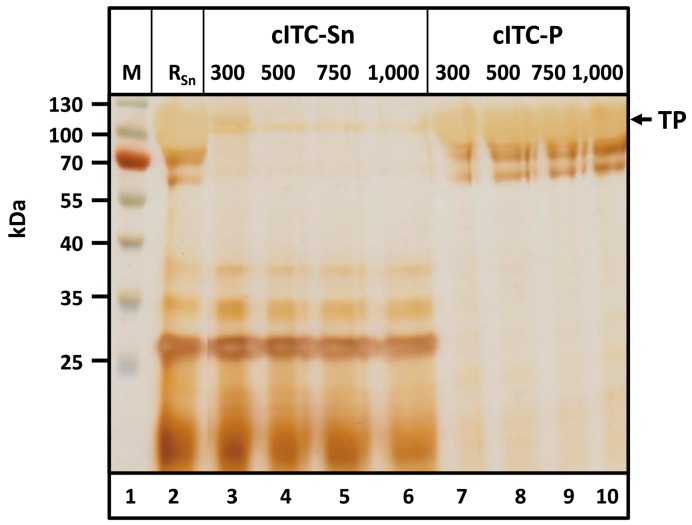
Polishing of Q-MaSp1-100× ELP by centrifugation-based Inverse Transition Cycling (cITC). A washed and lyophilized pellet resulting from fractionating acetone precipitation was resolubilized in 6 M urea and centrifuged. The supernatant (lane 2) was aliquoted and dialyzed against buffers containing 50 mM Tris-HCl (pH 8.0) and NaCl ranging from 300–1000 mM. Samples were then heated to 40 °C and centrifuged. The target protein (TP) was concentrated in the pellet (cITC-P), while soluble contaminants remained in the supernatant (cITC-Sn). M-Prestained protein ladder; *R*_Sn_-soluble fraction of resolubilized pellet after precipitation with 70% (*v*/*v*) acetone. The arrow indicates the molecular size of the target protein (TP).

After acetone treatment, precipitated proteins were resolubilized in 6 M urea, then dialyzed and precipitated at 40 °C to improve TP purity. Precipitates were removed by centrifugation. The NaCl concentration required to achieve heat-induced precipitation was determined via dialysis against buffers containing 300, 500, 750 and 1000 mM NaCl (Figure 5).

Solids remaining during resolubilization were removed via centrifugation. The supernatant (Figure 5, lane 2) contained large amounts of the TP. After cITC, the TP was found in the pellet (cITC-P, Figure 5, lanes 11–14). No HCPs were detected in the pellet for any of the NaCl concentrations investigated. At high protein concentrations, minor target protein degradation bands became visible between ~60 and 100 kDa. These degradation bands corresponded to the target protein, as verified by Western Blotting using an anti-c-myc-antibody and by mass spectrometry (LC-ESI-MS) (Figure S2 and Table S1). Proteinogenic contaminants remained soluble under the chosen conditions and were detected exclusively in the supernatant (cITC-Sn, Figure 5, lanes 7–10). At 300 mM NaCl, a relatively high amount of TP was detected in the supernatant. Pellets from cITC were dried and weighed. With 300, 500, 750 and 1000 mM NaCl, product masses were 20.1, 25.2, 27.5 and 27.0 mg, respectively. These data led to the conclusion that polishing of Q-/K-MaSp1-100× ELP via cITC should be performed using a buffer containing 50 mM Tris-HCl and 750 mM NaCl at pH 8.0.

### 2.5. Purification of ELPylated MaSp1 Proteins at Pilot Scale

**Figure 6:**
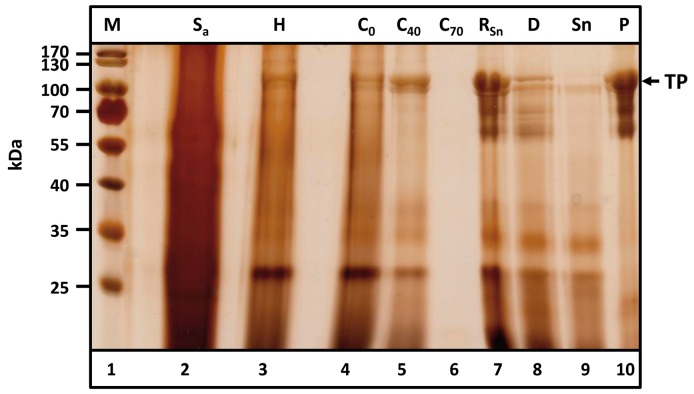
Pilot scale purification of Q-MaSp1-100× ELP from *Nicotiana tabacum* leaves. Samples of BWM2 (2 kg × 3 kg) were thawed (16 h, 4 °C), mixed with tap water (3 L, respectively) and disrupted (lane 2). The suspension was clarified via filtration (lane 3) and centrifugation (lane 4). Fractionating acetone precipitation was then performed by adjusting the acetone concentration first to 40% (C_40_—supernatant after centrifugation, lane 5) and then to 70% (C_70_—supernatant after centrifugation, lane 5). The resulting pellet was washed in tap water and lyophilized. Following resolubilization in 6 M urea and centrifugation, the supernatant (lane 7) was dialyzed against 50 mM Tris-HCl, 750 mM NaCl, pH 8.0 (lane 8). Finally, the target protein (TP) was separated from soluble contaminants via cITC (lanes 9 and 10). M, Prestained protein ladder; S_a_, Suspension a; H, Filtrate of the hydro press; C: Supernatants after centrifugation containing 0 (C_0_), 40 (C_40_) and 70% (*v*/*v*) (C_70_) acetone, respectively; R_Sn_, Soluble fraction of resolubilized pellet after precipitation with 70% (*v*/*v*) acetone; D, R_Sn_ dialyzed against 50 mM Tris-HCl with 750 mM NaCl at pH 8.0; Sn, Soluble fraction of the dialysis sample after cITC; P, Pellet of the dialysis sample after cITC. The arrow indicates the molecular size of the target protein (TP).

The purification procedure began with 5–6 kg tobacco leaves containing Q-MaSp1-100× ELP. The results of the consecutive steps are shown in Figure 6. Filtration of the suspension (Figure 6, lane 2) resulted in a slightly turbid filtrate (Figure 6, lane 3). Figure 6, lane 4 shows the clarified supernatant after centrifugation. The quality of the purified TPs remained almost constant. Precipitation with 40% (*v*/*v*) acetone yielded essentially the same results as for another batch in Figure 4 (Figure 6, lane 5). At 70% acetone, the TP is completely precipitated (Figure 6, lane 6, supernatant). Polishing of the TP was achieved by resolubilizing the washed pellet with subsequent centrifugation (Figure 6, lane 7), dialyzing the supernatant against 50 mM Tris-HCl with 750 mM NaCl at pH 8.0 (Figure 6, lane 8), and performing cITC at 40 °C. The TP was completely recovered from the pellet (Figure 6, lane 10). The supernatant contained only HCPs (Figure 6, lane 9). Therefore, the figure also shows, that HCPs are mainly separated from the target protein, thus showing a remarkable purity of spider silk protein preparations achieved by the methodology. The identity of the purified protein has been shown by LC-ESI-MS analysis of TP bands (Figure S2 and Table S1).

The described purification protocol was also performed for K-MaSp1-100× ELP. From 5.2 and 6.0 kg tobacco leaves, 273 mg K-MaSp1-100× ELP and 413 mg Q-MaSp1-100× ELP were isolated, respectively.

## 3. Discussion

The production of spider silk derivatives for biotechnical purposes requires efficient production systems and scalable and cost-effective purification methods. Plants offer several advantages for protein production for pharmaceutical use, including scalability and generally low contamination from pathogens [26]. A further advantage of plants is the low cost of infrastructure and production during the upstream phase [19]. The essential costs of plant-based molecular farming processes are incurred by downstream processing. In the case of plant-based biopharmaceuticals, downstream processing can account for up to 80% of overall production costs (for a review, see [19]).

In standard biotechnological processes, products are usually secreted by cells into the medium. Products can therefore be captured without disrupting cells [27]. Most efficient plant expression systems use targeting to intracellular compartments to allow for specific posttranslational modifications and to increase stability [28]. Retention in the Endoplasmic Reticulum has been the method of choice for many molecular farming applications [29]. Consequently, cell disruption including the breakage of cell walls is necessary to release proteins produced in whole plant tissues into solution [30]. A simple freeze-thaw cycle is usually insufficient for the isolation of proteins from tobacco leaves [31]. Therefore, mechanical disruption using blenders and homogenizers is the disruption method of choice [32,33,34].

Mean particle diameters obtained following disruption of tobacco leaves using a Waring blender at medium scale and a table top cutter at pilot scale were 1.8 µm and 1.5 µm, respectively (see Section 2). Comparable values were obtained using a polytron homogenizer [35]: particle sizes ranged from 1 to 10 µm and clogged the used bag filter. We used a conventional hydropress to clarify the resulting juice and did not observe clogging. However, a centrifugation step is recommended to fully remove particles.

Cell disruption liberates high concentrations of HCPs that then have to be removed. For structural proteins, the enzymatic activity does not need to be preserved. Therefore, low-tech methods, such as heat and solvent precipitation, may be used [36]. For instance, the production of gelatin from collagen is performed at temperatures above 45 °C [37]. The heat insensitivity of spider silk proteins is a remarkable property that has been successfully exploited for the enrichment of recombinant spider silk proteins from *Escherichia coli* (*E. coli*) [38,39] and transgenic plants [16]. The aims of heat treatment are the inactivation of proteases and the removal of HCPs by precipitation. Heat treatment has been successfully used for the purification of ELPylated hemagglutinin and ELPylated recombinant antibodies from leaves by mITC [40]. Heat treatment of plant leaves via blanching in a boiling solvent has been used to effectively remove HCPs during the purification of recombinant proteins produced in tobacco [35].

We investigated microwave heating as an alternative method to avoid large liquid volumes and to minimize the risk of TP loss. Provided that a microwave of appropriate size is available, this method is a very good alternative to blanching. Microwave treatment of the biomass is not only less time-consuming than blanching but also prevents TP loss due to leakage from disrupted cell walls.

Methods involving organic solvents are easy and scalable methods for the extraction and precipitation of proteins [41,42]. For instance, recombinant tropoelastin was purified from *E. coli* by solvent precipitation using isopropanol/butanol mixtures [41]. The solvent mixture used to produce tropoelastin was successfully replaced with acetone in our lab. Because the ELP-tag present at the C-terminus of our TP is based on the elastin sequence, we investigated whether the tropoelastin purification protocol could be adapted for the ELPylated MaSp1 analog. Optimization experiments were successfully performed at small scale, and optimal acetone concentrations were determined (see Results). These parameters were applied to the medium and pilot scales, and the major goal of producing soluble TPs using these conditions was achieved. Final recovery of the TP in 70% acetone allowed the separation of soluble HCPs by centrifugation.

During the scale up, the BWM:tap water ratio was decreased to 1:1 with no influence on TP purity after acetone precipitation. The amount of the product achieved by this method was astonishingly high (37–67 g from 5–6 kg leaf material).

Centrifugation-based Inverse Transition Cycling (cITC) is a powerful non-chromatographic process for the removal of remaining HCPs and inorganic salts. The ITC technique has been optimized for plants [43] and used for the purification of different ELPylated proteins from plants, including nanobodies [25], spider silk proteins [44] and hemagglutinins [15] (for a review, see [45]). The first challenge was to resolubilize the pellet after acetone precipitation, which was only possible using 6 M urea as a chaotropic agent. Dialysis was then used to remove urea and to determine the ionic strength necessary for cITC.

Minor TP degradation bands became visible when high amounts of spider silk protein were analyzed by SDS-PAGE. These degraded proteins were identified as product-related impurities using antibody detection Western blot and LC-ESI-MS (Figure S2 and Table S1). Precipitation behavior in the presence of acetone and during cITC of the truncated proteins was obviously identical to the intact TP. The origin of the truncated versions is not completely clear. These bands only became visible at very high concentrations in the final stage of the purification. During the different downstream processing steps, Western blots using antibodies were routinely used to check whether TP loss occurred. The truncated proteins are likely the result of mistranslations that were enriched during purification.

The upscaling of downstream processing is a major issue in plant molecular farming. Plants provide production systems that easily allow for upscaling. Non-chromatographic methods are suitable for increasing the scale of the process without significantly increasing downstream processing costs, which would prevent future product development [19]. The purification protocol we developed was scaled up to the 5–6 kg scale and used to purify two different (but closely related) proteins. The final product yield was 53 and 69 mg per kg BWM for lysine- and glutamine-tagged MaSp1-100× ELP, respectively. These results are in agreement with results from Weichert and co-workers, who reported yields of 65–75 mg product per kg leaf material [16].

The simple and scalable method for the purification of spider silk proteins from tobacco leaves that we have developed is a first step towards a production and downstream processing system that can provide materials for applications such as scaffolds for medical approaches or technical devices [46].

## 4. Materials and Methods

If not stated otherwise, all chemicals were purchased from Carl Roth GmbH (Karlsruhe, Germany).

### 4.1. Plant Material and Gowth Conditions

Strains of stably transformed transgenic *Nicotiana tabacum* cv. SNN containing gene expression cassettes for leaf-specific accumulation of glutamine- or lysine-tagged MaSp1-100× ELP monomers (Q-MaSp1-100× ELP and K-MaSp1-100× ELP) were recently described [16].

Transgenic tobacco seeds were cultured on Murashige-Skoog agar containing 50 mg/L kanamycin. Developing plants containing recombinant TPs (Q-MaSp1-100× ELP; K-MaSp1-100× ELP) were selected by immunoblotting leaf extracts using an anti-c-myc antibody [47]. Plants were then grown for 11 weeks in soil beds under glass without supplemental light or temperature regulation from May to July. Soil bed plants were fertilized with 0.3% Hakaphos blau (Available online: www.compo.de/) once a week. Before plants began to flower, tobacco leaf material was harvested 15 weeks after sowing.

### 4.2. Post-Harvest Treatment of Bio Wet Mass (BWM)

Tobacco leaves were treated in two different ways after plant defoliation. At the 50 g scale, leaves were frozen in liquid nitrogen and crushed using a masher (BWM1). At the 300 g scale, leaves were incubated in a microwave (NN-SD452W, Panasonic, Hamburg, Germany) at 1 kW for 7 min (BWM2). BWM1 and BWM2 were, respectively, stored at −80 and −20 °C until further use.

### 4.3. Cell Disruption at Small, Medium and Pilot Scales

At small scale (50 g BWM1), leaves were resuspended in 150 mL pre-heated (90 °C) disruption buffer (DB: 50 mM Tris-HCl, pH 8.0; [48]) and incubated for 1 h with >90 °C while gentle stirring. After incubation, leaves were disintegrated by mixing for 10 × 30 s at maximum power using a Waring blender (type 4184, Braun, Schwalbach am Taunus, Germany). At medium scale (300 g BWM2), leaves were thawed slowly for a minimum of 12 h at 4 °C and mixed with 450 mL cold tap water (4 °C) before disruption under the conditions described above. At the pilot scale (5–6 kg), leaves were disintegrated using a table top cutter R10 v.v. (Robot Coupe, Vincennes, France) for 5 min at maximum power after thawing for a minimum of 12 h at 4 °C and the addition of cold tap water at a ratio of 1:1 (*w*/*v*) BWM:tap water.

### 4.4. Fractionating Acetone Precipitation 

During the pre-experiment and following cell disruption, a 250-µL sample was transferred to an Eppendorf tube. Aliquots (1.0 mL) of acetone-dH_2_O-mixture containing increasing amounts of acetone were then added to adjust the acetone concentration of the solution from 0% to 70% (*v*/*v*) acetone. Finally, 1 mL pure acetone was added to adjust to the acetone concentration to 80% (*v*/*v*). After manual mixing and centrifugation (16, 100× *g*, 5 min, 4 °C) (Centrifuge 5415 R, Eppendorf AG, Hamburg, Germany), 1 mL of the supernatant was transferred to a new Eppendorf tube. The solvent was then evaporated (Eppendorf concentrator 5301, Eppendorf AG, Hamburg, Germany) for 12 h at 45 °C. Dried pellets were then dissolved in 440 µL SDS-PAGE sample buffer.

For all larger scales, the temperatures of the BWM1 and BWM2 cell suspensions were generally adjusted to 10 °C prior to acetone addition. Acetone concentrations were increased stepwise from 0% to 70% (*v*/*v*). After each stepwise addition, mixtures were incubated for 30 min at 4 °C. Samples were taken following each incubation step and centrifuged (16,100× *g*, 5 min, 4 °C). Based on the increasing dilution factor during fractionating acetone precipitation, sample volumes were continuously increased, beginning with 500 µL for the first precipitation step at the lowest acetone concentration. Collected supernatants were transferred into new Eppendorf tubes, then dried (Eppendorf concentrator 5301) for 12 h at 45 °C. Dried pellets were dissolved in 200 µL SDS-PAGE sample buffer.

The pilot scale cell suspension generated from 5–6 kg leaves was filtered using a hydropress (Hydropresse 20 L, Speidel GmbH, Ofterdingen, Germany) at 3 bar for 30 min. A mop tissue (Moptuch VILE143121, VWR International GmbH, Darmstadt, Germany) was used as the filtration membrane. The filter cake was washed with 1 L tap water, followed by a second filtration step under the same conditions as described above. Both filtrates were pooled, stored for 16 h at 6 °C and clarified by centrifugation (tubular centrifuge CEPA Z41G, Carl Padberg Zentrifugenbau GmbH, Lahr, Germany) with 10% pump power at 17,000 g. Acetone (40% (*v*/*v*)) was added to the clarified supernatant. After incubation at 6 °C for 30 min, the acetone mixture was centrifuged as described above. Acetone was then added to the supernatant to a final concentration of 70% (*v*/*v*). After centrifugation, the pellet was stored at −80 °C until further use.

### 4.5. Purification of Plant-Based ELPylated Spidroins by Centrifugal Inverse Transition Cycling (cITC)

Centrifugation-based Inverse Transition Cycling protein purification was performed according to Meyer and Chilkoti [24]. The cell suspension filtrate pellet obtained via fractionating acetone precipitation was washed with 6 volumes (*v*/*w*) of tap water for 3 h at 25 °C. The suspension was centrifuged at 17,700× *g* and 4 °C for 30 min (Avanti^®^ J-30I, rotor JA-10; Beckman Coulter GmbH, Krefeld, Germany). The resulting pellet was either lyophilized and resolubilized in 22 volumes of 6 M urea or directly resolubilized in 9 volumes of 6 M urea at 60 °C, then clarified via centrifugation at 40 °C and 3220× *g* for 15 min (Eppendorf centrifuge 5810R, swing bucket rotor A-4-81; Eppendorf AG, Hamburg, Germany). The supernatant was dialyzed against a 100 volume of dialysis buffer (50 mM Tris-HCl, pH 8.0 with 300, 500, 750, or 1000 mM NaCl) four times at 4 °C for 2 h in dialysis tubing (Spectra/Por^®^7, MWCO 15 kDa; Carl Roth GmbH, Karlsruhe, Germany). Dialysates were warmed to 40 °C in a water bath and centrifuged at 3220× *g* and 40 °C for 30 min (Eppendorf centrifuge 5810R, swing bucket rotor A-4-81). The pellet was lyophilized (Lyovac GT 2-E; Steris GmbH, Huerth, Germany) for further downstream analysis.

### 4.6. SDS-PAGE and Protein Detection

Samples were dried in a SpeedVac (Eppendorf Concentrator 5301) for at least 8 h at 45 °C, resolubilized in sample buffer (0.2 M Tris-HCl; 5% (*w*/*v*) SDS; 10% (*w*/*v*) glycerol; 0.05% (*w*/*v*) Bromophenol-blue; pH 6.8; 1% (*v*/*v*) β-mercaptoethanol) and boiled at 99 °C for 5 min. Proteins were separated by reducing SDS-PAGE [49] and either electro-transferred to nitrocellulose membranes (Whatman GmbH, General Electric Deutschland Holding GmbH, Frankfurt am Main, Germany) using 25 mM Tris-HCl, 0.1% (*w*/*v*) SDS, 192 mM glycine and 20% (*v*/*v*) methanol for Western analysis or visualized by silver staining according to [50] or stained by Coomassie^®^ Brillant Blue R-250 (SERVA Electrophoresis GmbH, Heidelberg, Germany). Western immunodetection was performed as described by [51] using anti-c-myc (9E10) supernatant [47] followed by horseradish peroxidase (HRP)-conjugated sheep anti-mouse immunoglobulin G (IgG) (GE Healthcare UK Ltd., Fairfield, UK). Proteins were visualized using enhanced chemiluminescence (Amersham ECL Plus TM, GE Healthcare UK Ltd., Fairfield, UK) or using SigmaFast™ BCIP/NBT (#B5655, Sigma-Aldrich Chemie GmbH, München, Germany). Prestained protein ladder was purchased from ThermoFisher Scientific (#26616; Thermo Fisher Scientific, Waltham, MA, USA).

### 4.7. Liqiud Chromatopraphy-Electrospray Ionization-Mass Spectrometry (LC-ESI-MS)

In-gel trypsin digested protein bands were cut out of gels and incubated for 45 min at 50 °C with 10 mM dithiothreitol (Sigma-Aldrich Chemie GmbH, München, Germany) in 100 mM ammonium bicarbonate. After removal of liquid, gel pieces were incubated in 55 mM iodoacetamide (Sigma-Aldrich Chemie GmbH, München, Germany) for 45 min in the dark to modify cysteine residues. The liquid was removed, and gel pieces were washed twice with 100 mM ammonium bicarbonate and then with 100 mM ammonium bicarbonate in 50% acetonitrile. Gel pieces were dried, reswollen in 20 µL 50 mM ammonium bicarbonate (pH 8.0) and digested with trypsin (Promega GmbH, Mannheim, Germany) overnight at 37 °C. For ESI-QTOF-MS/MS analysis peptides were extracted from gel pieces and injected into a nanoACQUITY UPLC system equipped with a binary solvent manager, sample manager and a heating and trapping module. Samples (2 µL) were injected in “microliter pickup” mode and desalted on-line using a symmetry C18 180 µm × 20 mm precolumn. Peptides were separated on a 100 µm × 100 mm analytical RP column (1.7 µm BEH 130 C18) using a typical UPLC gradient from 3.0% to 33.0% over 15 min. The mobile phases used were 0.1% formic acid in water and 0.1% formic acid in acetonitrile. The column was connected to an SYNAPT^®^ G2 HDMS mass spectrometer.

The SYNAPT G2 HDMS is a hybrid quadrupole tandem time-of-flight (Q-TOF) mass spectrometer. Data were acquired in LC/MSE mode, switching between low and elevated energy on alternate scans. Subsequent correlation of precursor and product ions was then achieved using both retention and drift time alignment. All parts were purchased from Waters GmbH, Germany. BiopharmaLynx (1.3.2, Waters GmbH, Eschborn, Germany) was used to analyze the obtained MS data.

## 5. Conclusions

**Figure 7:**
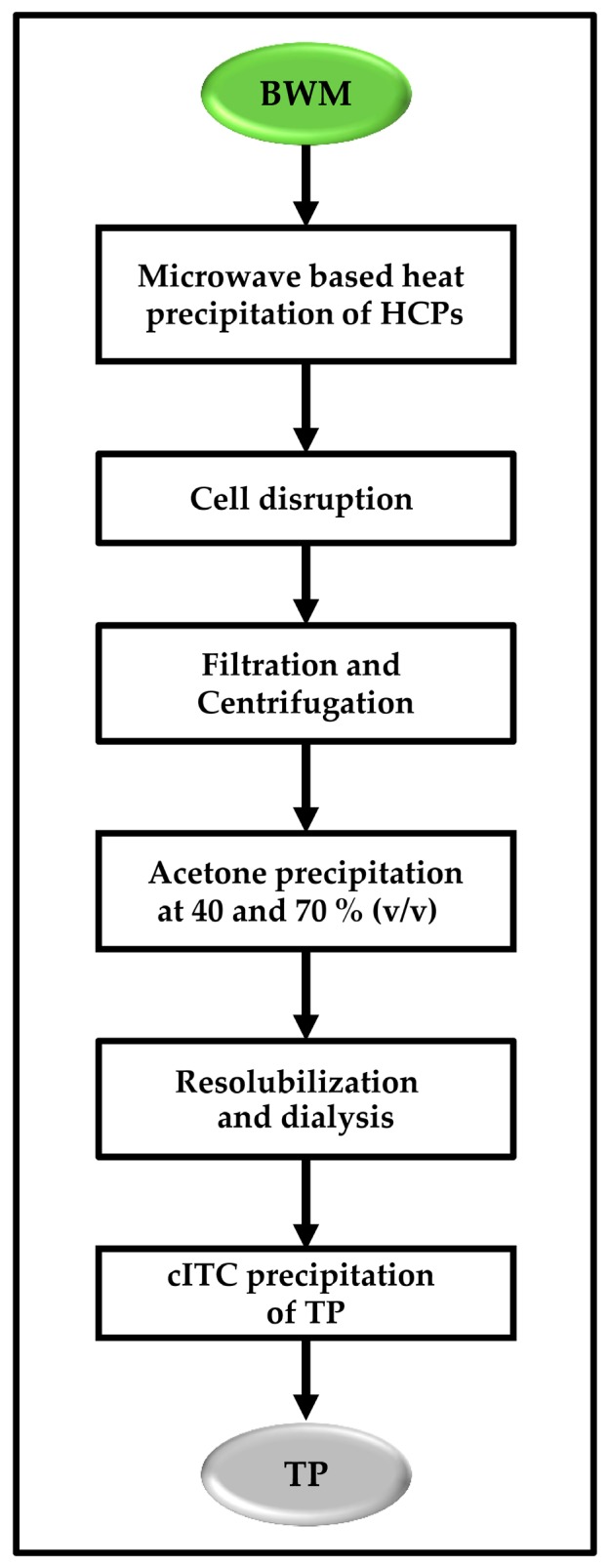
Low-tech pilot scale purification scheme for the capture and polishing of a spider silk analog. Up to 6 kg BWM were processed. In total, 413 mg Q-MaSp1-100× ELP and 273 mg K-MaSp1-100× ELP were isolated.

A plant-based expression system is used to produce spider silk protein derivatives. The further use of spider silk proteins for the production of non-woven tissues or for the coating of scaffolds for tissue engineering needs the purification of these proteins in a scalable manner at low cost. Heat treatment, acetone precipitation and Inverse Transition Cycling are used in a cheap, low-tech and scalable downstream processing strategy (see Figure 7). This procedure can be adapted for the purification of other structural proteins, including additional spider silk analogs, elastin, and collagen.

## Supplementary Materials

Supplementary materials can be found at www.mdpi.com/1422-0067/17/10/1687/s1.
