# Supplementary material for: Low-Tech, Pilot Scale Purification of a Recombinant Spider Silk Protein Analog from Tobacco Leaves
Source: Int J Mol Sci. 2016 Oct 9;17(10):1687. doi: 10.3390/ijms17101687 (PMC5085719; doi:10.3390/ijms17101687)
Supplement: Supplementary file 1 [file ijms-17-01687-s001.pdf]

Supplementary Material: Low-Tech, Pilot Scale Purification of a Recombinant Spider Silk Protein Analog from Tobacco Leaves

René Heppner, Nicola Weichert, Angelika Schierhorn, Udo Conrad and Markus Pietzsch

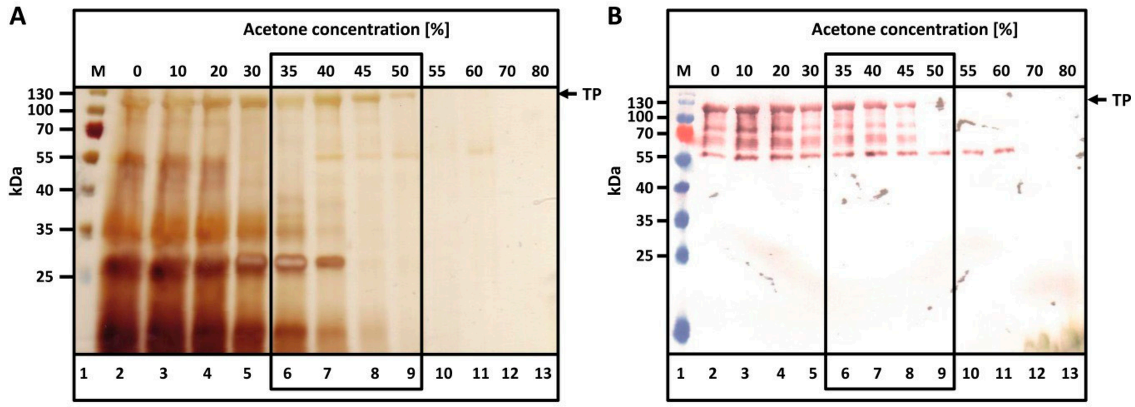

Figure S1. Pre-experiment for fractionating acetone precipitation. In 250 µL suspension of disrupted tobacco leaves (BWM1) in disruption buffer the respective acetone concentration was adjusted by mixing with an acetone-dH2O-mixture (1.0 mL). After incubation (30 min, 4 °C) the suspension was centrifuged and 1 mL of the supernatant was dried. Following, the dry content was resolubilized in 440 µL SDS-PAGE sample buffer and boiled (5 min, 99 °C), and the samples were analyzed via SDS-PAGE. Marked with a rectangle is the range of acetone concentration which was investigated in the small scale experiment. TP-target protein (K-MaSp1-100× ELP); (**A**) Silver stained gel; (**B**) Western Blot. The arrow indicates the molecular size of the target protein (TP).

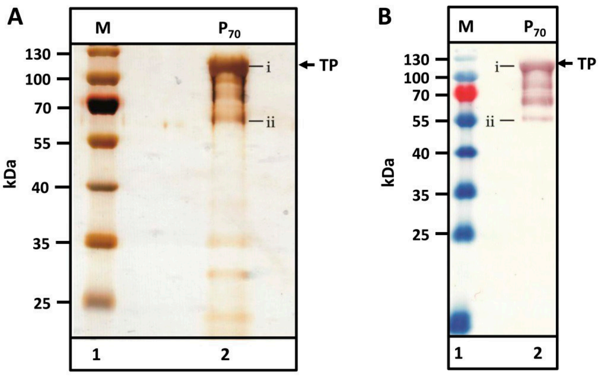

Figure S2. LC-ESI-MS samples. After SDS-PAGE, two bands (i and ii) were cut out of after detection on a Coomassie stained gel. Following, a tryptic digestion was performed, and the resulting sample was analyzed via LC-ESI-MS. TP-target protein (K-MaSp1-100× ELP); (**A**) Silver stained gel; (**B**) Western Blot; M-Prestained protein ladder; P_{70}-Resulting pellet after precipitation at 70% (*v*/*v*) acetone. The arrow indicates the molecular size of the target protein (TP).

Table S1. LC-ESI-MS analysis of purified K-MaSp1-100× ELP. Two samples (i) and (ii) corresponding to two bands on an SDS-PAGE gel (see Figure S1) were applied to a nanoACQUITY UPLY system after tryptic digestion. Within the whole protein sequence of the target protein K-MaSp1-100× ELP [16], found peptides are shown bold. Peptides which directly connected to each other in the sequence were separated by ± underlining.

(i)
SGPGAAQGAGAAAAAAAGGAGQGGYGGLGGQGAGQGGYGGLGGQGAGQGAGAAAAAAAGGAGQGGY<br>GGLGSQGAGR**GGQGAGAAAAAAAGGAGQGGYGGLGSQGAGRGGLGGQGAGAAAAAAAGGAGQGGYGGLG**<br>**NQGAGRGGQGAAAAAAAGGAGQGGYGGLGSQGAGR**GGLGGQGAGAAAAAAAGGAGQGGYGGLGG<br>QGAG**QGGYGGLGSQGAGRGGLGGQGAGAAAAAAAGGAGQGGLGGQGAGQGAGASAAAAGGAGQGGYGG**<br>**LGSQGAGR**GGEGAGAAAAAAAGGAGQGGYGGLGGQGAG**QGGYGGLGSQGAGR**GGLGGQGAGAA<br>AAGGAGQGGLGGQGAGQGAGAAAAAAAGGAG**QGGYGGLGSQGAGR**GGLGGQGAGAVAAAAAGGAGQGGYGG<br>LGNQGAGR**GGQGAAAAAAGGAGQGGYGGLGSQGAGRGGQGAGAAAAAAVGAGQGGYGGLGGQGAGQGGY**<br>**GGLGSQGSGRGGLGGQGAGAAAAAAAGGAGQGGLGGQGAGQGAGAAAAAAAGGVRQGGYGGLGSQGAGRG**<br>**GQGAGAAAAAAAGGAGQGGYGGLGGQGVGRGGLGGQGAGAAAAAGGAGQGGYGGVGSGASAASAAASRLSS**<br>**PQASSR**LSSAVSNLVATGPTNSAALSSTISNVVSQIGASNPGLSGCDVLIQALLEVVSALIQILGSSSIGQVNYGSAGQATQ<br>IVGQSVYQALGVYQALGAGGQAAAEQKLISEEDLNGAVEMGHGVGVPGVGVPGGGVPGAGVPGVGVPGVGVPGVG<br>VPGGGVPGAGVPGGGVPGVGVPGVGVPG<br>GVPGAGVPGVGVPGVGVPGVGVPGGGVPGAGVPGGGVPGVGVPGVGVPGGGVPGAGVPGVGVPGVGVPGVGVPG<br>GGVPGAGVPGGGVPGVGVPGVGVPGGGVPGAGVPGVGVPGVGVPGVGVPGGGVPG<br>AGVPGGGVPGVGVPGVGVPGGGVPGAGVPGVGVPGVGVPGVGVPGGGVPGAGVPGGGVPGVGVPGVGVPGGGVP<br>GAGVPGVGVPGVGVPGVGVPGGGVPGAGVPGGGVPGVGVPGVGVPGGGVPGAGVPG<br>VGVPGVGVPGVGVPGGGVPGAGVPGGGVPGVGVPGVGVPGGGVPGAGVPGVGVPGVGVPGVGVPGGGVPGAGVP<br>GGGVPGVGVPGVGVPGGGVPGAGVPGVGVPGVGVPGVGVPGGGVPGAGVPGGGVPG<br>VGVPGVGVPGGGVPGAGVPGVGVPGVGVPGVGVPGGGVPGAGVPGGGVPGGLAAAEPKDEL
(ii)
SGPGAAQGAGAAAAAAAGGAGQGGYGGLGGQGAGQGGYGGLGGQGAGQGAGAAAAAAAGGAGQGGY<br>GGLGSQGAGR**GGQGAGAAAAAAAGGAGQGGYGGLGSQGAGRGGLGGQGAGAAAAAAAGGAGQGGYGGLG**<br>**NQGAGRGGQGAAAAAAAGGAGQGGYGGLGSQGAGR**GGLGGQGAGAAAAAAAGGAGQGGYGGLGG<br>QGAG**QGGYGGLGSQGAGR**GGLGGQGAGAAAAAAAGGAGQGGLGGQGAGQGAGASAAAAGGAG**QGGYGGLG**<br>**SQGAGR**GGEGAGAAAAAAAGGAGQGGYGGLGGQGAG**QGGYGGLGSQGAGR**GGLGGQGAGAA<br>AAGGAGQGGLGGQGAGQGAGAAAAAAAGGAG**QGGYGGLGSQGAGR**GGLGGQGAGAVAAAAAGGAGQGGYGG<br>LGNQGAGR**GGQGAAAAAAGGAGQGGYGGLGSQGAGRGGQGAGAAAAAAVGAGQGGYGGLGGQGAGQGGY**<br>**GGLGSQGSGRGGLGGQGAGAAAAAAAGGAGQGGLGGQGAGQGAGAAAAAAAGGVRQGGYGGLGSQGAGRG**<br>**GQGAGAAAAAAAGGAGQGGYGGLGGQGVGRGGLGGQGAGAAAAAGGAGQGGYGGVGSGASAASAAASRLSS**<br>**PQASSR**LSSAVSNLVATGPTNSAALSSTISNVVSQIGASNPGLSGCDVLIQALLEVVSALIQILGSSSIGQVNYGSAGQATQ<br>IVGQSVYQALGVYQALGAGGQAAAEQKLISEEDLNGAVEMGHGVGVPGVGVPGGGVPGAGVPGVGVPGVGVPGVG<br>VPGGGVPGAGVPGGGVPGVGVPGVGVPG<br>GGVPGAGVPGVGVPGVGVPGVGVPGGGVPGAGVPGGGVPGVGVPGVGVPGGGVPGAGVPGVGVPGVGVPGVGVP<br>GGGVPGAGVPGGGVPGVGVPGVGVPGGGVPGAGVPGVGVPGVGVPGVGVPGGGVPG<br>AGVPGGGVPGVGVPGVGVPGGGVPGAGVPGVGVPGVGVPGVGVPGGGVPGAGVPGGGVPGVGVPGVGVPGGGVP<br>GAGVPGVGVPGVGVPGVGVPGGGVPGAGVPGGGVPGVGVPGVGVPGGGVPGAGVPG<br>VGVPGVGVPGVGVPGGGVPGAGVPGGGVPGVGVPGVGVPGGGVPGAGVPGVGVPGVGVPGVGVPGGGVPGAGVP<br>GGGVPGVGVPGVGVPGGGVPGAGVPGVGVPGVGVPGVGVPGGGVPGAGVPGGGVPG<br>VGVPGVGVPGGGVPGAGVPGVGVPGVGVPGVGVPGGGVPGAGVPGGGVPGGLAAAEPKDEL
